# Antibacterial Biopolymer Gel Coating on Meshes Used for Abdominal Hernia Repair Promotes Effective Wound Repair in the Presence of Infection

**DOI:** 10.3390/polym13142371

**Published:** 2021-07-20

**Authors:** Selma Benito-Martínez, Bárbara Pérez-Köhler, Marta Rodríguez, Francisca García-Moreno, Verónica Gómez-Gil, Gemma Pascual, Juan Manuel Bellón

**Affiliations:** 1 Departamento de Medicina y Especialidades Médicas, Universidad de Alcalá, Ctra. Madrid-Barcelona Km. 33.600, Alcalá de Henares, 28805 Madrid, España; selma.benito@uah.es (S.B.-M.); barbara.perez@uah.es (B.P.-K.); 2 Biomedical Networking Research Centre on Bioengineering, Biomaterials and Nanomedicine (CIBER-BBN), 28029 Madrid, Spain; marta.rodriguezma@uah.es (M.R.); francisca.garciam@uah.es (F.G.-M.); veronica.gomezg@uah.es (V.G.-G.); juanm.bellon@uah.es (J.M.B.); 3 Ramón y Cajal Health Research Institute (IRYCIS), 28034 Madrid, Spain; 4 Departamento de Cirugía, Ciencias Médicas y Sociales, Universidad de Alcalá, Ctra. Madrid-Barcelona Km. 33.600, Alcalá de Henares, 28805 Madrid, España; 5 Departamento de Ciencias Biomédicas, Universidad de Alcalá, Ctra. Madrid-Barcelona Km. 33.600, Alcalá de Henares, 28805 Madrid, España

**Keywords:** antimicrobial, biopolymers, chlorhexidine, hernia, mesh coating, mesh infection, rifampicin, tissue repair

## Abstract

Prosthetic mesh infection is a devastating complication of abdominal hernia repair which impairs natural healing in the implant area, leading to increased rates of patient morbidity, mortality, and prolonged hospitalization. This preclinical study was designed to assess the effects on abdominal wall tissue repair of coating meshes with a chlorhexidine or rifampicin-carboxymethylcellulose biopolymer gel in a *Staphylococcus aureus* (*S. aureus*) infection model. Partial abdominal wall defects were created in New Zealand white rabbits (*n* = 20). Four study groups were established according to whether the meshes were coated or not with each of the antibacterial gels. Three groups were inoculated with *S. aureus* and finally repaired with lightweight polypropylene mesh. Fourteen days after surgery, implanted meshes were recovered for analysis of the gene and protein expression of collagens, macrophage phenotypes, and mRNA expression of vascular endothelial growth factor (VEGF) and matrix metalloproteinases (MMPs). Compared to uncoated meshes, those coated with either biopolymer gel showed higher collagen 1/3 messenger RNA and collagen I protein expression, relatively increased VEGF mRNA expression, a significantly reduced macrophage response, and lower relative amounts of MMPs mRNAs. Our findings suggest that following mesh implant these coatings may help improving abdominal wall tissue repair in the presence of infection.

## 1. Introduction

The repair of an abdominal wall defect using a prosthetic material is today a common surgical procedure with some 20 million surgical interventions conducted annually worldwide [1]. Although the use of a prosthetic mesh has shown clear benefits for patients, complications associated with the implant of foreign material include seromas [2], mesh migration [3], mesh infection [4], mesh fistula [5], and chronic pain [6] that can arise postoperatively.

The incidence of prosthetic mesh infection, which could be as high as 13%, depends on several factors such as the type of hernia pathology, the surgical procedure and the implanted prosthesis [7]. In effect, prosthetic mesh infection is considered one of the most devastating post-surgical complications, and its occurrence has been associated with increased rates of patient morbidity and mortality, prolonged hospitalization, and additional costs to the healthcare system [4]. In hernia surgery, the main types of microorganisms responsible for prosthetic material infection are *Staphylococcus aureus* (*S. aureus)* and *S. epidermidis* [4]. The presence of microorganisms in the implanted mesh and surrounding tissues negatively affects the normal tissue repair process. Infection increases the risk hernia recurrence determining that a second surgery will be required. This problem has turned the attention of researchers to the extracellular matrix (ECM). So far, several ECM alterations, especially those related to collagen metabolism and matrix degrading metalloproteinases (MMPs), have been described [8,9].

Collagens are the most abundant component of the ECM. Immature type III collagen, predominantly found in the early stages of wound healing, is replaced by mature type I collagen during this physiological process [10]. In effect, a proper ratio of synthesized and deposited collagens type I and type III is essential for connective tissue remodeling. In fibroblasts isolated from the fascia and skin of patients with incisional and inguinal hernia, several studies have shown there is a reduced ratio of type I to type III collagen [7,11,12].

In response to the tissue injury produced by the implant of a prosthetic material in the abdominal wall, macrophages start to produce and secrete different types of cytokines and growth factors. Among others, vascular endothelial growth factor (VEGF) seems to be an important promotor of angiogenesis, which plays an essential role in wound healing and host tissue incorporation in the implant [13].

In in vitro and in vivo preclinical studies in rabbit models, we found that coating polypropylene meshes with gel compounds loaded with either chlorhexidine [14] or rifampicin [15] reduced the prevalence of *S. aureus* on the implant surface. Similarly, other studies conducted in rats have detected antibacterial efficacy against *Escherichia coli* of polyvinyl fluoride (PVDF) meshes coated with a gentamicin-loaded polymer along with an increased collagen type I/III ratio at the mesh–host tissue interface and lowered expression of collagenases [16].

Macrophages play a key role in the foreign body reaction to an implanted prosthetic mesh for hernia repair. These cells can show either a proinflammatory M1 phenotype or an anti-inflammatory M2 phenotype. M1 macrophages play an essential role in acute inflammation and produce characteristic proinflammatory cytokines such as TNF-α, lL-1b, IL-6, IL-12, and IL-23 and expression of markers such as CD80 and CD86 [17]. In addition, M1 macrophages are critical for host protection against viruses and intracellular bacteria during acute infections. In contrast, M2 macrophages promote tissue remodeling and fibrosis, produce ECM components and angiogenic factors, and increase the expression of several markers such as IL-10, CD163, CD206, arginase 1, MHC-II, TGM2, TGF-β, and IL-1RA [17,18]. The balance between M1 and M2 macrophages plays an important role in the phagocytosis of pathogens, the clearance of apoptotic cells and the healing and remodeling of injured tissues [19].

MMPs belong to a family of enzymes involved in remodeling and degrading the ECM and basement membranes. MMP-2 is necessary for proper wound healing and is essential for appropriate angiogenesis, inflammation, and fibrosis [20]. Olaso et al. demonstrated close correlation between collagen formation and degradation and MMP-2 expression during wound repair [21]. Further, in prior work we observed MMP-2 upregulation in the abdominal skin of patients with direct inguinal hernia [22]. It has also been observed that MMP-9 contributes to ECM remodeling and the substrates of this metalloproteinase include denatured collagens, type IV collagen, type V collagen, elastin, fibronectin, and modulates VEGF release [23]. Recent studies have shown increased MMP-9 expression in a mouse model of skin infection [24] and MMP-2 gene transcription modulation in gentamicin-coated meshes which optimized their integration within the abdominal wall [25].

Within this context, the aim of this preclinical study was to examine the effect of mesh infection on wound healing in terms of collagen deposition, VEGF and MMP expression and the macrophage phenotypes elicited in the implant zone. Once this effect of infection was established, we assessed the impact on this wound repair process of the use of an antibacterial biopolymer gel-coated mesh.

## 2. Materials and Methods

### 2.1. Experimental Animals and Ethics

Twenty male, New Zealand White rabbits of mean weight 3000 g were used. The study was carried out in accordance with the Guide for the Care and Use of Laboratory Animals of the National and European Institutes of Health (Spanish law 06/2013, Spanish Royal Decree 53/2013, European Directive 2010/63/UE and European Convention of the Council of Europe ETS123). All procedures were performed at the University’s Animal Research Center which is registered with the Directorate General for Agriculture of the Council of Economy and Technology Innovation of the Community of Madrid (ES280050001165) indicating that all facilities legally cover the needs and requirements of the research. The study protocol was approved by the Committee on the Ethics of Animal Experiments of the University of Alcalá.

### 2.2. Prosthetic Material

The biomaterial used in this study was Optilene Mesh Elastic (B. Braun, Melsungen, Germany). This is a reticular lightweight (48 g/m^2^) polypropylene mesh with a pore surface area of 7.64 ± 0.32 mm^2^ designed for the repair of abdominal wall defects. The mesh was cut into 5 × 2 cm fragments under sterile condition.

### 2.3. Antibacterial Biopolymer Gel Coatings

Antibacterial chlorhexidine gluconate (CHX) gel was elaborated as described elsewhere [14]. Rifampicin (RIF) 0.03% *v/v* gel (Sigma-Aldrich, St. Louis, MO, USA), was elaborated following the same procedures. The gels were prepared the day before the assays under sterile conditions and stored protected from light at 4 °C.

### 2.4. Scanning Electron Microscopy and UV-Vis Biopolymer Gels Characterization

For scanning electron microscopy (SEM), mesh fragments (1 × 1 cm^2^) were coated by immersion in the formulated gels (*n* = 3 each) under sterile conditions and the coating was air-dried overnight at room temperature, in a Telstar AV 30/70 type-II vertical laminar flow hood (Telstar S.A., Madrid, Spain). Dried samples were placed onto aluminium pin stubs, metalized with gold palladium and examined in a JSM-IT500 InTouchScope™ SEM (JEOL Ltd., Akishima, Tokio, Japan). Uncoated meshes (*n* = 3) were included as control.

Spectrophotometric analyses were performed to record the UV–Vis absorption spectra of CHX- and RIF-loaded biopolymer gels. To determine the spectrum of the gel carrier, measurements were also performed in drug-free 1% CMC. Freshly prepared gels were allowed to stabilize for 24 h at room temperature prior to carry out the measurements, which were recorded in triplicate. An Ultrospec 3100 Pro spectrophotometer (Amersham Biosciences, Little Chalfont, UK) with a wavelength accuracy of 1 nm, a 10 mm matched quartz cell and in the spectral range of 200 to 800 nm, was used. To avoid exceeding the detection threshold of this technique, RIF-gel concentration was diluted (1:3) in CMC 1%.

### 2.5. Bacterial Inocula

The bacterial strain used to inoculate the implants was *S. aureus* ATCC25923 (Spanish Type Culture Collection; CECT, Valencia, Spain). Immediately before surgery, bacterial suspensions containing approximately 1–1.5 × 10^6^ CFU/mL were prepared by spectrophotometry, as described elsewhere [14].

### 2.6. Experimental Design

The 20 animals were randomly distributed among the different study groups, established according to the type of mesh coating and their corresponding control uncoated and uninfected meshes. Each device was treated by immersion in either the CHX-loaded or RIF-loaded gel for 5 min immediately before its implant. The study groups were as follows:

- Control (infection control): uncoated meshes without infection.
- Uncoated (coating control): uncoated meshes inoculated with *S. aureus*.
- CHX: meshes coated with the CHX-loaded gel inoculated with *S. aureus*.
- RIF: meshes coated with the RIF-loaded gel inoculated with *S. aureus*.

In our prior work [14,15], no evidence of cytotoxicity, systemic drug diffusion, nor host tissue alterations was observed when antiseptic or antibiotic coatings were tested. Because of this and to fulfill animal well-being criteria (3Rs: replacement, reduction, and refinement) we did not include a group of meshes coated with CHX or RIF without *S. aureus* infection.

### 2.7. Surgical Technique

**Figure 1:**
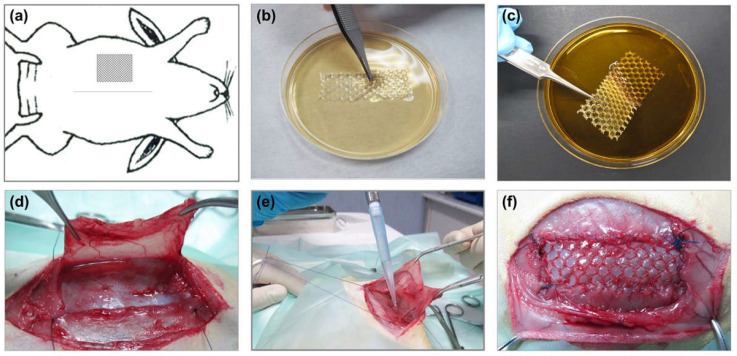
Mesh coating procedures and surgical technique. (**a**) Diagram illustrating the surgical procedure. Mesh coating by immersion in (**b**) chlorhexidine or (**c**) rifampicin biopolymer gel. (**d**) Partial hernia defects (5 × 2 cm) created in the right anterior side of the abdominal wall. (**e**) *S. aureus* inoculation in the surgical defect. (**f**) Detail of an implanted mesh.

Animals were anesthetized with a combination of ketamine (20 mg/kg, Imalgene, Merial, Barcelona, Spain) and xylazine (3 mg/kg, Xilagesic 2%, Calier, Barcelona, Spain) administered intramuscularly. Analgesic dose was administrated as described elsewhere [14,15]. Using a sterile surgical technique, partial hernia defects (5 × 2 cm) were created in the right anterior side of the abdominal wall, according to the protocol developed by our group [26]. The *S. aureus* suspension was prepared according to a method described elsewhere [14]. Mesh was secured to the margins of the defect and skin tissue was closed as described elsewhere [14,15] (Figure 1). After 14 days of implant, the animals were sedated with an anesthetic cocktail and then euthanized with a lethal dose of 20% sodium pentobarbital (Dolethal, Vetoquinol SA, Lure, France), according to the guidelines for the euthanasia of experimental animals. Immediately following euthanasia, implants were harvested, cut into several fragments and processed as described elsewhere [14].

### 2.8. Histological Study

The procedures used for light microscopy have been described elsewhere [14]. Samples were visualized using a Zeiss Axiophot light microscope (Carl Zeiss, Oberkochen, Germany).

### 2.9. RNA Isolation and Quantitative Real-Time Polymerase Chain Reaction (qRT-PCR)

Fragments of the implants (1 cm^2^ in size) containing mesh plus neoformed host tissue were obtained and stored at −80 °C until further analysis. For qRT-PCR, total RNA was isolated using TRIzol reagent (Invitrogen, Carlsbad, CA, USA) by means of the guanidine-phenol–chloroform isothiocyanate procedure. The amount and purity of RNA were determined in a NanoDrop ND-1000 spectrophotometer (Thermo Fisher Scientific Inc., Waltham, MA, USA). Complementary DNA (cDNA) was synthesized from 200 ng of total RNA by reverse transcription (RT) using oligo dT primers (Amersham, Fairfield, CT, USA) and the M-MLV reverse transcriptase enzyme (Invitrogen, Carlsbad, CA, USA). cDNA was amplified using the following rabbit primer sequences: collagen 1A2 (sense 5′-ATG GTG GCA CCC AGT TTG AA-3′ and antisense 5′-AGG TGA TGT TCT GAG AGG CG-3′); collagen 3A1 (sense 5′-TGC TAA GGG TGA AGT TGG AC-3′ and antisense 5′-CCG CCA GGA CTA CCA TTG TT-3′); VEGF (sense 5′-GGA GTA CCC TGA TGA GAT CGA-3′ and antisense 5′-CTT TGG TCT GCA TTC ACA TTT GT-3′); MMP-2 (sense 5′-CCT TCA ACT GGA GCA AGA-3′ and antisense 5′-TCT TCT TCT TCA CCT CAT TGT A-3′); and MMP-9 (sense 5′-TAC CGA GAG AAA GCC TAC and antisense 5′-CTG GTC CAC TAG GTT CAC-3′).

To identify the different macrophage phenotypes, cDNA was amplified using specific primers for M1 macrophages: tumor necrosis factor alpha (TNF-α) (sense 5′-CTC CTA CCC GAA CAA GGT CA -3′ and antisense 5′-CGG TCA CCC TTC TCC AAC T-3′), cluster of differentiation 80 (CD80) (sense 5′-GCA CTG TCC TGT GAT TAC-3′ and antisense 5′- CAT CTG TTG GTC CTT CTG-3′) and cluster of differentiation 86 (CD86) (sense 5′-AAT GGA TAA GGC AGA GAA TG-3′ and antisense 5′-AAC GAT GTT CAC ACT TGG-3′); and for M2 macrophages: interleukin-10 (IL-10) (sense 5′-GAA CTC CCT GGG GGA AAA C-3′ and antisense 5′-GGC TTT GTA GAC GCC TTC CT-3′) and mannose receptor C-Type 1 (MRC1, CD206) (sense 5′-TGA TGG GAC CCC TGT AAC CT-3′ and antisense 5′-TGC CCA GTA TCC ATC CTT GC-3′). The housekeeping gene glyceraldehyde 3-phosphate dehydrogenase (GAPDH) (sense 5′-TCA CCA TCT TCC AGG AGC GA-3′ and antisense 5′-CAC AAT GCC GAA GTG GTC GT-3′) was used as an internal control.

qRT-PCR was performed in a StepOnePlus Real-Time PCR System (Applied Biosystems, Foster City, CA, USA). The samples were analyzed in triplicate and gene expression was normalized against the expression value recorded for the constitutive gene GAPDH. The products were subjected to 2% agarose gel electrophoresis, stained with a SYBR Green II RNA gel stain (Invitrogen, Carlsbad, CA, USA) and visualized under UV light.

### 2.10. Immunofluorescence Analysis of Collagens

The expression of collagen I and collagen III at the protein level was examined by immunofluorescence labeling with the monoclonal antibodies anti-collagen I (Sigma-Aldrich, St. Louis, MO, USA) and anti-collagen III (Medicorp, Montreal, Canada). Anti-mouse-rhodamine-conjugated antibody was used as secondary antibody (Jackson ImmunoResearch, Suffolk, UK). Cell nuclei were counterstained with 4,6-diamidino-2-phenylindole (DAPI). Immunofluorescence was detected using a Leica SP5 confocal microscope (Leica Microsystems, Wetzlar, Germany) through the Confocal Microscopy Service (ICTS ‘NANBIOSIS’ U17) of the Biomedical Research Networking Centre on Bioengineering, Biomaterials and Nanomedicine (CIBER-BBN at the University of Alcalá, Madrid, Spain).

### 2.11. Statistical Analysis

Data were expressed as the mean and standard error. To compare different study groups, the Mann-Whitney U test was used. All statistical tests were performed using the GraphPad Prism 5 computer package (GraphPad Software Inc., La Jolla, CA, USA). Significance was set at *p* < 0.05.

## 3. Results

### 3.1. In Vitro Characterization of Biopolymer Gels

**Figure 2:**
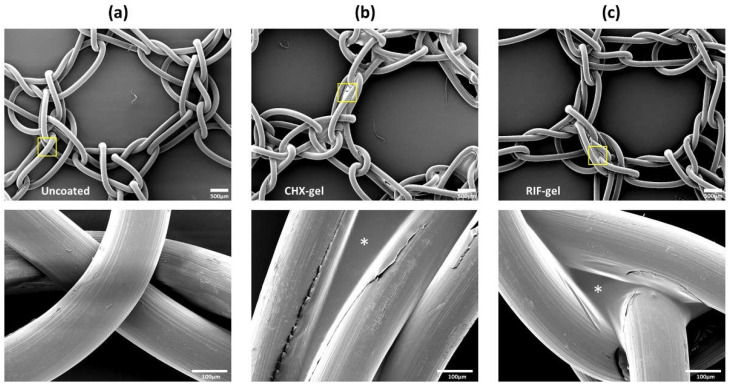
Characterization of the formulated biopolymer gels by scanning electron microscopy (SEM). (**a**) Uncoated meshes, meshes coated with (**b**) CHX or (**c**) RIF biopolymer gel (magnification ×200; scales: 500 µm). To provide a better visualization of biopolymer gels (*), some areas (boxes) were magnified (×2000; scales: 100 µm).

SEM observations showed that uncoated meshes presented a knitted polypropylene monofilament creating large pores. Once coated, CHX and RIF biopolymer gels were found covering the prosthetic filaments without occluding the mesh pores. Some of the micropores created by the interweaving of the filament also showed polymeric coating (Figure 2).

**Figure 3:**
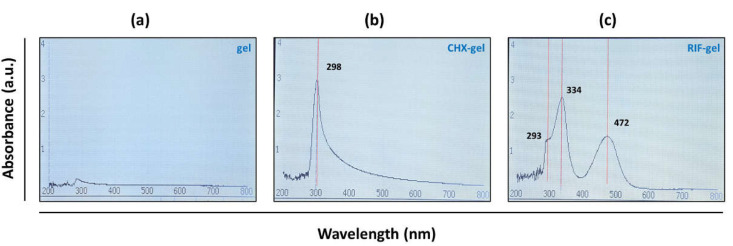
Characterization of (**a**) unloaded gel, (**b**) CHX, and (**c**) RIF formulated biopolymer gels by UV-Vis absorption spectra.

With respect to UV-Vis analysis, the spectrum of CHX-gel showed absorbance at 298 nm wavelength with 2.9 absorbance value on average. The UV spectrum of RIF-gel showed absorbance at three wavelengths, namely, 293 nm, 334 nm, and 472 nm with corresponding specific average absorbance values 1.32, 2.52, and 1.45, respectively (Figure 3). These data are in agreement with the findings of others [27,28], who observed UV–Vis spectra of rifampicin displaying several peaks distributed at similar wavelengths than those recorded by us.

### 3.2. Histological Findings

**Figure 4:**
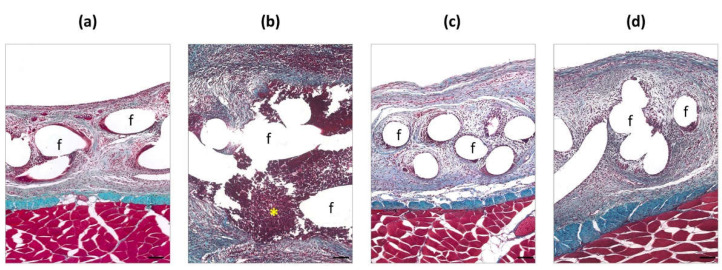
Histological findings in (**a**) control (non-infected), (**b**) uncoated, (**c**) CHX-, and (**d**) RIF-coated implants. For each group, micrographs illustrate host tissue incorporation into the meshes (Masson’s trichrome, ×100). Scale bars: 100 µm. Symbols: (**f**) mesh filaments; (*) abscess.

Fourteen days after surgery (Figure 4), control uninfected meshes presented adequate integration within the host tissue. Mesh pores were infiltrated by a neoformed loose connective tissue and inflammatory cells were observed around the polypropylene filaments.

In contrast, abscesses of *S. aureus*, embedded in a dense neoformed connective tissue, were observed in the uncoated meshes inoculated with bacteria. In those zones, these displayed poor integration within the host tissue and revealed the presence of inflammatory cells around the material. Observations from the CHX and RIF groups were similar to those in the uninfected controls, showing a completely integrated mesh within a loose connective tissue that infiltrated the mesh pores with no microscopic signs of infection.

### 3.3. Collagens Gene Expression (qRT-PCR)

**Figure 5:**
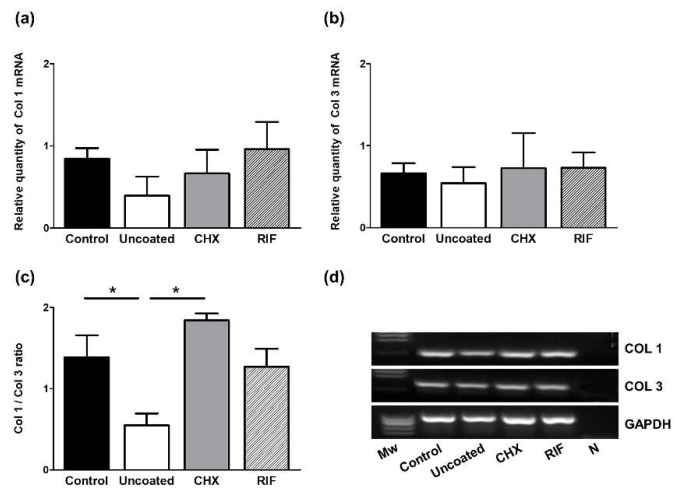
(**a**) Relative mRNA levels of collagen 1, (**b**) collagen 3, and (**c**) Col 1/Col 3 ratio in the different experimental groups determined by qRT-PCR in the implant areas. Gene expression was normalized to expression recorded for the reference gene GAPDH. Values are expressed as the mean ± SEM. (**d**) Agarose gel electrophoresis of RT-PCR products. Molecular markers (*M*w) and negative control (N). Mann-Whitney U test: * *p* < 0.05.

The gene expression of collagens 1 and 3 was examined in the neoformed tissue in areas close to the polypropylene implants. Although no significant differences were detected among the different groups for collagen 1 mRNA expression, lowest levels were detected in the uncoated inoculated meshes, while the other groups showed the similar expression of collagen 1 mRNA (Figure 5a).

A similar trend was observed in all the study groups for collagen 3 mRNA expression levels (Figure 5b). However, significant differences were observed in the Col 1/Col 3 ratio in that the uncoated mesh group showed a significantly lower ratio than both the uninfected control and CHX groups (*p* < 0.05). Meshes in the RIF group also showed an increased Col 1/Col 3 ratio compared to the uncoated ones, although the difference was not significant (Figure 5c).

### 3.4. Protein Expression of Collagens

**Figure 6:**
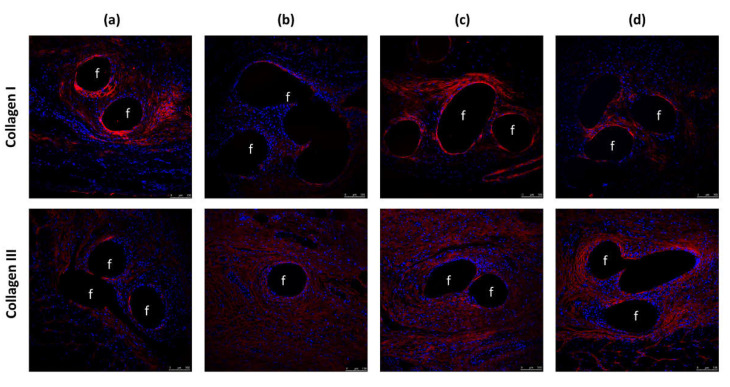
Immunofluorescence labeling for collagen I and III protein in (**a**) control (non-infected), (**b**) uncoated, (**c**) CHX-, and (**d**) RIF-coated implants. Neoformed collagen appears in red, and cell nuclei (stained with 40,6-diamidino-2-phenylindole (DAPI)) appear in blue. Symbols: (**f**) mesh filaments.

In an immunofluorescence analysis, we examined the production and deposition of collagens type I and type III in the different implants (Figure 6). In general, for the uncoated group, the intensity of labeled-collagen I was lower than in the other groups. The expression of mature collagen was moderate in the RIF group. Similar results were obtained in the CHX group, while control non-infected meshes showed greater collagen I deposits. In all the groups, collagen I expression was mainly localized around the prosthetic filaments. The intensity of collagen III labeling was reduced in the uncoated infected meshes, whereas in the remaining groups, this intensity was similar and localized in the neoformed tissue throughout the implanted mesh.

### 3.5. VEGF Gene Expression (qRT-PCR)

**Figure 7:**
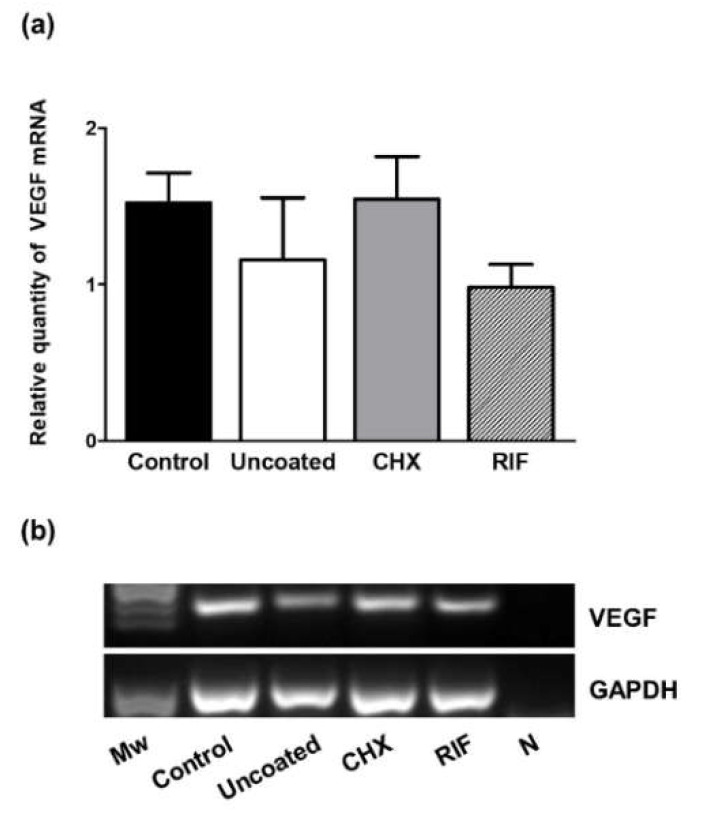
(**a**) Relative mRNA levels of VEGF in the different experimental groups were determined by qRT-PCR in the implant areas. Gene expression was normalized to the expression recorded for the reference gene GAPDH. Values are expressed as the mean ± SEM. (**b**) Agarose gel electrophoresis of RT-PCR products. Molecular markers (*M*w) and negative control (N).

Levels of VEGF mRNA expression were examined by qRT-PCR (Figure 7). Although no significant differences emerged among groups, VEGF mRNA expression observed in both the control and CHX implants were similar, this expression being higher compared to observations in the uncoated or RIF meshes.

### 3.6. M1/M2 Macrophages (qRT-PCR)

To assess the macrophage response to the different implants, several biomarkers related to the proinflammatory (M1) or reparative (M2) phenotypes of these cells were assessed by qRT-PCR.

**Figure 8:**
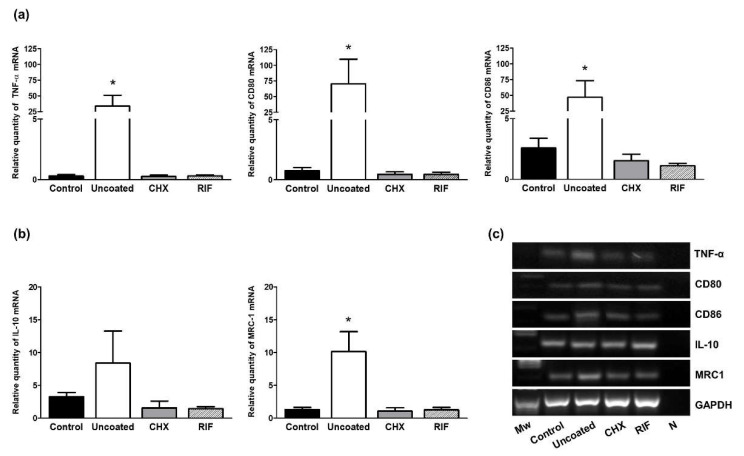
Relative mRNA levels determined by qRT-PCR in the implant areas. (**a**) TNF-α, CD80, and CD86 (M1 macrophage markers) mRNA levels. (**b**) IL-10 and MRC1 (M2 macrophage markers) mRNA levels. Gene expression was normalized to expression recorded for the reference gene GAPDH. (**c**) Agarose gel products. Molecular markers (*M*w) and negative control (N). Mann–Whitney U test: * *p* < 0.05.

Similar expression patterns were observed of genes coding for the M1 phenotype markers (TNF-α, CD80, and CD86), with higher relative amounts of these mRNAs detected in the uncoated meshes compared to the control uninfected meshes and those coated with CHX or RIF (*p* < 0.05) (Figure 8a).

In addition, the uncoated meshes showed higher mRNA expression levels for the different genes associated with the M2 phenotype (MRC1 and IL-10) compared to the remaining groups, although the relative quantity of expression of these two markers was lower than that of M1 yet significant differences only emerged for MRC1 mRNA (*p* < 0.05) (Figure 8b).

### 3.7. Expression of MMPs (qRT-PCR)

**Figure 9:**
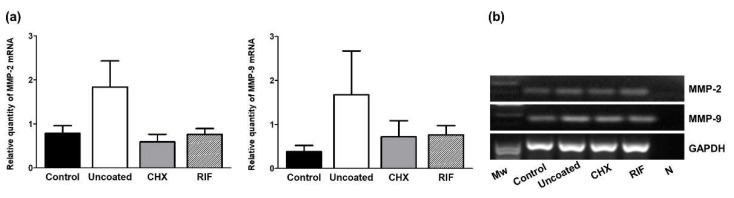
(**a**) Relative mRNA levels of metalloproteinases (MMP-2 and MMP-9) were determined by qRT-PCR in the implant areas in the different experimental groups. Gene expression was normalized to the expression recorded for the reference gene GAPDH. Values are expressed as the mean ± SEM. (**b**) Agarose gel electrophoresis of RT-PCR products. Molecular markers (*M*w) and negative control (N). Mann–Whitney U test: * *p* < 0.05.

Our MMP expression data (Figure 9) indicate that both mRNAs for MMP-2 and MMP-9 were more expressed in the uncoated group, than in the other groups, but without significance between them.

## 4. Discussion

Since the introduction of polypropylene mesh for the repair of abdominal hernia defects [29], the use of biomaterials has been a breakthrough in this type of surgery. However, despite its advantages, the implantation of a biomaterial in the abdominal wall can give rise to a series of complications, some of them related to bacterial mesh infection.

Prosthetic infection is one of the most significant complications following mesh implant. In the majority of cases, a second surgery to remove the implant is needed, thus increasing rates of morbidity, mortality and hospital stay with the corresponding impacts on healthcare costs.

Bacterial infection can modify the natural tissue remodeling process. In this milieu, components of the ECM play a critical role in wound healing. The main protein that provides a basic scaffold of neoformed connective tissue is collagen. Collagen type I is mature, mechanically stable, and is known to provide tensile strength, whereas collagen type III is immature and mechanically unstable. The proportions of collagen types I and III determine the mechanical stability of connective tissue [30]. Patients with inguinal and incisional hernia show a reduction in the collagen type I/III ratio [31,32]. In addition, in a study examining the reasons for the explant of 78 meshes such as infection, chronic pain and hernia recurrence, a lower type I/III collagen ratio was observed in meshes removed because of hernia recurrence compared to those explanted because of chronic pain or infection [33]. A possible explanation for hernia recurrence is an abnormal balance between type I and type III collagen due to insufficient scar formation. However, very few studies have addressed the effects of mesh infection in terms of the quantity and quality of collagen formation.

Our results indicate lower levels of mRNA for collagen I in the uncoated meshes infected with *S. aureus* compared to the control and prophylactic meshes in which bacterial infection was prevented. As confirmed in our immunofluorescence study, the outcome of such diminished mRNA levels is a decrease in RNA–protein translation and consequently the reduced synthesis and deposition of collagen protein at this short-term time point after mesh implantation. In contrast, while we observed similar amounts of collagen III mRNA in all the implants, the uncoated meshes showed a significantly reduced type I/III collagen ratio, which indicates a delay in the formation of repair tissue.

Other studies have established animal models of postoperative mesh infection similar to the *S. aureus* one used here [14,15]. The antibacterial efficacy of meshes coated with a polymer loaded with gentamicin [34], vancomycin [35], and ofloxacin [36] has also been reported. In prior work, we assessed the use of a carboxymethylcellulose gel loaded with the antiseptic chlorhexidine [14] or the antibiotic rifampicin [15]. Our findings confirmed the effectiveness of both prophylactic strategies to avoid infection following hernia repair surgery. However, few studies have examined the effects of these prophylactic coatings on collagen synthesis and neoformed tissue deposition on the hernial defect.

The data presented here reveal the increased expression of type I collagen mRNA in the prophylactic-coated meshes with respect to the uncoated meshes, with the consequent increased collagen type I/III ratio in these implants. This higher ratio points to a more mature neoformed tissue, which could confer strength to the repair zone. In the presence of the chlorhexidine coating, this ratio was similar to that observed in the control meshes. However, when the coating contained rifampicin, this ratio was lower. Further, we observed a greater amount of collagen I in the chlorhexidine-coated meshes mainly surrounding the mesh filaments. These data are in agreement with the findings of Junge et al. [16], who noted a higher collagen I/III ratio in PVDF meshes supplemented with gentamicin leading to improved scar quality and host tissue incorporation.

Another issue we considered was the effect of mesh infection on angiogenesis. VEGF is a powerful inducer of angiogenesis which is essential in the early proinflammatory response and for the synthesis, deposition, and organization of a new ECM [13]. MMPs are responsible for the degradation of several ECM components and also play a significant role in angiogenesis in the proximity of wounds. Interestingly, although some studies have shown that MMP-9 and MMP-2 can stimulate VEGF expression [37,38], the results of our study indicate that in uncoated infected meshes mRNA levels of VEGF decreased while MMP-9 and MMP-2 mRNA levels were upregulated. Additionally, we observed these effects were reversed in the case of the prophylactic-coated meshes. Our findings are in line with those of other studies in which higher VEGF and lower MMP expression were observed in a mouse model of wounds infected with methicillin-resistant *S. aureus* treated with the antibiotics dalbavancin [24] or teicoplanin [39].

Macrophages are involved in several processes such as host defense, tissue homeostasis, and the foreign body reaction induced by the implant of a biomaterial. Macrophages may be polarized across a spectrum between two functional phenotypes: the classically activated proinflammatory M1 phenotype, associated with host defense and foreign body reaction, and the activated M2 phenotype, associated with reparative tissue remodeling [40]. In previous preclinical studies [14,15], we observed an improved macrophage response when an antimicrobial mesh was implanted in the abdominal wall. However, we did not discriminate between M1 and M2 phenotypes. The present data revealed that the presence of *S. aureus* infection in uncoated meshes provokes an elevated M1-macrophage response at 14 days postimplant, showing significant differences with respect to the other groups. These results are consistent with the findings of others [41] who reported that the presence of bacterial lipopolysaccharides and/or proinflammatory cytokines stimulates the activation of M1-macrophages, implicated in both the inflammatory and antimicrobial response. Our analysis of the M2 macrophage phenotype by qRT-PCR revealed that the uncoated meshes showed the increased gene expression of M2 cell population markers. In the context of tissue remodeling, we expected to find this M2 increase in the groups overcoming *S. aureus* infection with a higher collagen I/III mRNA ratio. However, we did not observe this modulation as wound healing is not complete within 14 days after surgery. Madsen et al. found that through a mannose receptor (MRC1), M2 macrophages are responsible for intracellular collagen degradation in vivo [42]. These findings are consistent with our results indicating this receptor is significantly increased in the uncoated meshes which showed less collagen expression.

To our knowledge, little information exists about how *S. aureus* modifies the macrophage response in vivo. However, the increase in M2 macrophages in our uncoated meshes, where *S. aureus* infection was established, is also consistent with findings in a short-term mouse model, in which the authors observed that *S. aureus* biofilms can re-program the macrophage response towards a pro-fibrotic M2 phenotype [43].

The main limitation of our study was its duration. When designing the experimental model, we established a single follow-up time of 14 days postsurgery mainly focused on assessing abdominal wall tissue repair when the acute prosthetic infection was in the process of consolidation. This could explain why we were unable to find macrophage phenotype changes which would occur in the longer term. Studies are needed to examine these implants over longer periods of time to monitor processes of tissue remodeling and macrophage responses on prophylactic meshes, as well as the impact that bacterial infection exerts on ECM quality.

## 5. Conclusions

The findings of this preclinical study indicate that the prophylactic antibacterial coating of a hernia repair mesh has beneficial effects both in terms of controlling infection and promoting tissue repair in the infected surgical implant zone.

## Data Availability

The data presented in this study are available in the manuscript.
